# Comparison of an ordinal endpoint to time-to-event, longitudinal, and binary endpoints for use in evaluating treatments for severe influenza requiring hospitalization

**DOI:** 10.1016/j.conctc.2019.100401

**Published:** 2019-06-21

**Authors:** Ross L. Peterson, David M. Vock, Abdel Babiker, John H. Powers, Sally Hunsberger, Brian Angus, Armando Paez, James D. Neaton, for the INSIGHT FLU-IVIG study group

**Affiliations:** a University of Minnesota School of Public Health, Division of Biostatistics, Minneapolis, MN, USA; b MRC Clinical Trials Unit at University College London, London, UK; c Collaborative Clinical Research Branch (CCRB), National Institute of Allergy and Infectious Diseases, Rockville, MD, USA; d Biostatistics Research Branch (BRB), National Institute of Allergy and Infectious Diseases, Rockville, MD, USA; e Nuffield Department of Medicine, Oxford University, Oxford, UK; f Division of Infectious Diseases, Baystate Medical Center, University of Massachusetts Medical School – Baystate, Springfield, MA, USA

**Keywords:** Clinical trials, Endpoints, Outcome assessments, Proportional odds model, Statistical power

## Abstract

**Background/aims:**

The Food and Drug Administration recommends research into developing well-defined and reliable endpoints to evaluate treatments for severe influenza requiring hospitalization. A novel 6-category ordinal endpoint of patient health status after 7 days that ranges from death to hospital discharge with resumption of normal activities is being used in a randomized placebo-controlled trial of intravenous immunoglobulin (IVIG) for severe influenza (FLU-IVIG). We compare the power of the ordinal endpoint under a proportional odds model to other types of endpoints as a function of various trial parameters.

**Methods:**

We used closed-form analysis and empirical simulation to compare the power of the ordinal endpoint to time-to-event, longitudinal, and binary endpoints. In the simulation setting, we varied the treatment effect and the distribution of the placebo group across the follow-up period with consideration of adjustment for baseline health status.

**Results:**

In the analytic setting, ordinal endpoints of high granularity provided greater power than time-to-event endpoints when most patients in the placebo group had either naturally progressed to the category of hospital discharge by day 7 or were far from hospital discharge on day 7. In the simulation setting, adjustment for baseline health status universally raised power for the proportional odds model. Across different placebo group distributions of the ordinal endpoint regardless of adjustment for baseline health status, only time-to-event endpoints yielded higher power than the ordinal endpoint for certain treatment effects.

**Conclusions:**

In this case study, the FLU-IVIG ordinal endpoint provided greater power than time-to-event, binary, and longitudinal endpoints for most scenarios of the treatment effect and placebo group distribution, including the target population studied for FLU-IVIG. The ordinal endpoint was only surpassed by the time-to-event endpoint when many patients in the placebo group were on the cusp of hospital discharge on day 7 and the follow-up period for the time-to-event endpoint was extended to allow for additional events. Our general approach for evaluating the power of several potential endpoints for an influenza trial can be used for designing other influenza trials with different target populations and for other trials in other disease areas.

## 1 Introduction

Among patients with severe influenza requiring hospitalization, no trial has demonstrated substantial clinical efficacy of an antiviral drug [1]. The United States Food and Drug Administration (FDA) recommends that primary endpoints in randomized controlled trials evaluating new treatments for patients hospitalized with influenza include any of the following measures: clinical symptoms, duration of hospitalization, time-to-normalization of vital signs, requirements for supplemental oxygen, and mortality. In this setting, FDA guidance recommends research into developing well-defined and reliable endpoints that have clinically meaningful outcomes for patients [1].

Following a successful pilot study of intravenous hyperimmune immunoglobulin (IVIG) [2], the International Network for Strategic Initiatives in Global HIV Trials (INSIGHT) launched a placebo-controlled trial of IVIG (FLU-IVIG) to evaluate its efficacy in hospitalized influenza patients (NCT02287467) [3]. A 6-category ordinal scale of patient health status measured 7 days after randomization serves as the primary endpoint for FLU-IVIG. The ordinal endpoint draws from both observed and self-reported outcome assessments to construct categories ranked in subsequent order of patient health status. A recent report by a working group from the U.S. Department of Health and Human Services advocates the use of an ordinal endpoint like the one in FLU-IVIG [4]. Two other randomized trials are using an ordinal scale as either the primary or a secondary endpoint (NCT02572817 & NCT03376321) [5,6].

Many factors must be considered when selecting an endpoint for a trial, including clinical relevance, potential bias in ascertainment, and statistical power. In a previous paper, we examined a number of design assumptions that may affect power for the FLU-IVIG ordinal endpoint under the pre-specified proportional odds model, including deviations from proportional odds, misclassification between the subjective categories of oxygen use or not and discharged or not, number of categories, and the anticipated distribution of the ordinal endpoint in the placebo group [7]. In this paper, we compare the power of the ordinal endpoint to other clinically relevant endpoints that were also considered, including time-to-event, longitudinal, and binary endpoints.

## 2 Methods

### 2.1 The INSIGHT FLU-IVIG trial

The FLU-IVIG study is a multicenter, double-blind randomized trial comparing IVIG versus placebo in hospitalized patients with locally confirmed influenza A or B who have a National Early Warning Score of two or higher [8]. Patients receive IVIG or placebo in addition to standard of care treatment. The primary endpoint for FLU-IVIG is the following 6-category ordinal outcome evaluated 7 days after randomization:

1) death;
2) intensive care unit (ICU) hospitalization;
3) non-ICU hospitalization, requiring supplemental oxygen;
4) non-ICU hospitalization, not requiring supplemental oxygen;
5) discharged from the hospital but unable to resume normal activities;
6) discharged from the hospital with resumption of normal activities.

The categories were defined to delineate clinically relevant change in patient health status due to IVIG. Day 7 was chosen for evaluation because a pilot study had established that differences in influenza antibody titer levels between IVIG and placebo were highest in the first few days following randomization [2]. The ordinal endpoint was chosen over a binary endpoint (e.g., proportion of patients discharged by day 7) because it was thought to provide more power and clinical information about patient recovery. Note that the ordinal endpoint ignores patient health trajectory across follow-up (e.g., hospitalized patients are counted as equal to re-admitted patients), only evaluating status on day 7.

The FLU-IVIG protocol specifies that the estimated odds ratio from fitting a proportional odds cumulative logistic model will be used to evaluate the effect of IVIG. Under the proportional odds assumption of the model, the odds ratios for any of the five better versus worse divisions of the ordinal endpoint (e.g., discharged versus hospitalized or dead) are constant. That is, the effect of IVIG is no more likely to benefit patients in one category versus another. Even if the proportional odds assumption is violated, the estimated odds ratio is still a valid measure of treatment efficacy for hypothesis testing and can be interpreted as the average shift across the ordinal endpoint due to IVIG, or alternatively as the odds of having a more favorable outcome due to IVIG compared to placebo. Note that the estimated odds ratio is not the arithmetic mean of the odds ratio for every possible binary division of the ordinal endpoint but is instead a nonlinear function of the probabilities of each category of the ordinal endpoint in the placebo and IVIG groups. The score test of the odds ratio is equivalent to the Wilcoxon rank-sum test [9].

With consideration for the anticipated distribution of the ordinal endpoint in the placebo group, the FLU-IVIG trial had a sample size of 320 patients to detect an odds ratio of 1.77 with 80% power at the 0.05 (two-sided) level of significance. An odds ratio greater than 1 indicates a more favorable outcome due to IVIG.

### 2.2 Analytic comparison of ordinal endpoint to time-to-event endpoint

We analytically compare the power of an ordinal endpoint to a time-to-event endpoint both derived from the same information. For concreteness, we refer to the time-to-event endpoint as time-to-hospital discharge, where deaths are censored at the end of follow-up and first hospital discharge counts as the event. The power for most hypothesis tests at the 0.05 (two-sided) level of significance is approximately equal to:

Power≅Φ(−1.96+c)

where Φ denotes the cumulative density function of the standard normal distribution and c is the non-centrality parameter which varies based on the type of endpoint chosen and the data generating mechanism.

We assume that time-to-hospital discharge follows an accelerated failure time (AFT) model with an exponential distribution and constant hazard ratio between treatment groups [10]. The AFT model assumes that the treatment proportionally increases or decreases the quantiles (e.g., median) of the duration of hospitalization. The non-centrality parameter for the AFT model, *c*_*a*_, is given by (see supplementary material for derivation):

$$c_{a}=\sqrt{n}\;*\;\log\left[\frac{\text{log}\left(1-p_{1t}\right)}{\text{log}\left(1-p_{0t}\right)}\right]*\left[\frac{1}{\sqrt{2}}{\left(\frac{1}{p_{0t}}+\frac{1}{p_{1t}}\right)}^{-1/2}\right],$$

where n denotes the total sample size assuming equal randomization to both groups, log denotes the natural logarithm, and $p_{it}$ denotes the probability of discharge by the end of follow-up (i.e., categories 5 and 6 of the ordinal endpoint combined) for the ith randomized group (0 denotes placebo, 1 denotes treatment) assuming a follow-up period of t days. We assume the same follow-up period for all patients because in influenza trials which motivate this work, follow-up is typically short and endpoints such as survival and hospital discharge are frequently assessed with very little missing data. Because the FLU-IVIG trial protocol expects minimal missing data on day 7 and time-to-hospital discharge is easily recorded, we assumed no missing data in both the analytic and simulation settings.

We assume that the ordinal endpoint follows a proportional odds model, as specified in the FLU-IVIG trial. For the proportional odds model, the non-centrality parameter, *c*_*p*_, under the proportional odds assumption on day 7 is provided by Whitehead [11] and is approximately equal to:

$$c_{p}≅\sqrt{n}*\log\left[\frac{p_{17}\left(1-p_{07}\right)}{p_{07}\left(1-p_{17}\right)}\right]*\sqrt{\frac{\left(1-\sum_{i=1}^{k}{\bar{q}}_{i}^{3}\right)}{12}},$$

where ${\bar{q}}_{i}$ denotes the average categorical probability between both randomized groups for the ith category of an ordinal endpoint with *k* categories, and the fraction $\frac{p_{17}\left(1-p_{07}\right)}{p_{07}\left(1-p_{17}\right)}=1.77$ from FLU-IVIG. The term $1-\overset{k}{\underset{i=1}{\sum }}{\bar{q}}_{i}^{3}$ measures the granularity of the ordinal endpoint with larger values indicating an ordinal endpoint whose category proportions are more evenly spread. For example, assume that we have an ordinal endpoint with three categories. If patients in the placebo group are evenly spread about the categories with proportions (1/3, 1/3, 1/3), and the distribution of patients in the treatment group meets proportional odds (odds ratio = 1.77 from FLU-IVIG) with category proportions (0.22, 0.31, 0.47), then $1-\overset{k}{\underset{i=1}{\sum }}{\bar{q}}_{i}^{3}=0.88$. Conversely, if patients in the placebo group tend to fall towards the last category with (1/10, 1/10, 4/5), and the treatment group again meets proportional odds (odds ratio = 1.77) with (0.06, 0.06, 0.88), then $1-\overset{k}{\underset{i=1}{\sum }}{\bar{q}}_{i}^{3}=0.41$.

Because both non-centrality parameters are proportional to $\sqrt{n}$, this implies that relative comparisons of the two models according to power do not depend on sample size for sufficiently large n. We compared the power of both endpoints according to different values of $1-\overset{k}{\underset{i=1}{\sum }}{\bar{q}}_{i}^{3}$ for the ordinal endpoint and different follow-up periods for the time-to-event endpoint. We varied $1-\overset{k}{\underset{i=1}{\sum }}{\bar{q}}_{i}^{3}$ as it measures the granularity of the ordinal endpoint, which is what a trial designer would want to maximize (e.g., by splitting or collapsing categories to be more evenly spread) to raise power. We fixed follow-up at day 7 for the ordinal endpoint in accordance with the FLU-IVIG trial and because in a trial with longer follow-up than 7 days, the two hospital discharge categories may be more difficult to ascertain. We extended the follow-up period of the time-to-event endpoint from 7 to 14 days to allow for more time for the event of hospital discharge to occur. Thus, we aimed to make a comparison between the ordinal and time-to-event endpoints under follow-up periods considered optimal for each. For both endpoints, we additionally varied the probability of discharge by day 7 ($p_{07}$) as a measure of the underlying risk of the population enrolled in the study. All other factors (e.g., treatment effect odds ratio of 1.77) were held constant. Note that we assume that hospital discharge constitutes at least one category of the ordinal endpoint; thus, values of $1-\overset{k}{\underset{i=1}{\sum }}{\bar{q}}_{i}^{3}$ below $1-\;{\left(\frac{p_{07}+p_{17}}{2}\right)}^{3}$ are not possible.

### 2.3 Simulation comparison of ordinal endpoint to other endpoints

Closed form expressions for (approximate) power only exist for a small number of endpoints. Furthermore, these expressions typically assume that the analysis model is consistent with the data generating mechanism. To broaden the scope of our study, we used simulation to compare the ordinal endpoint to other types of endpoints for data that does not follow a constant hazard ratio between treatment groups. We considered six different endpoints each evaluated on day 7, the pre-specified time point for FLU-IVIG:

• E1: Proportion of patients hospitalized or dead on day 7.
• E2: Proportion of patients moving to less severe categories from day 0 to day 7.
• E3: Winners versus losers between IVIG and placebo on day 7.
• E4: Day 7 ordinal endpoint.
• E5: Longitudinal measures of the ordinal endpoint over days 1–7 of follow-up.
• E6: Time-to-first hospital discharge.

We considered seven models to fit to these six endpoints. We fitted a simple logistic regression model to both E1 and E2, but given that E2 compares patient status for two different time points, we refer to its fitted model as the sliding dichotomy [12]. The endpoint E3 considers all possible comparisons of patients in the IVIG group to those in the placebo group according to their given ordinal endpoint category on day 7 [13]. The summary measure for this endpoint, called the win ratio, calculates the number of comparisons of greater health status for IVIG relative to placebo (wins) divided by the number of comparisons of worse health status (losses). Without any stratification, the win ratio performs similar to the well-known nonparametric Wilcoxon rank-sum test [14].

We fitted the proportional odds model to E4 and a longitudinal ordinal outcome model to E5 using generalized estimating equations assuming an independent working correlation matrix [15]. The longitudinal ordinal outcome model includes a term for treatment group, day of assessment (treated as a continuous variable), and day by treatment group interaction to model the distributions of the ordinal endpoint in both randomized groups over days 1–7 of follow-up. The day by treatment group interaction captures the treatment effect as the average multiplicative change in the odds ratio from the proportional odds model across days. For example, if the treatment effect proportionally increases by day up to an odds ratio of 1.77 on day 7, then the coefficient for the day by treatment group interaction would be 1.085 such that 1.085^7^ = 1.77.

We fitted both the Cox proportional hazards model and the AFT model to E6 [16]. Across the follow-up period, the Cox model calculates the hazard ratio of hospital discharge between groups. To allow for more flexibility in modeling time-to-hospital discharge for the AFT model, we assumed a Weibull distribution in addition to an exponential distribution. All seven models with their corresponding six endpoints are displayed in Table 1. In addition to models which only include the treatment effect, we considered models which adjusted for baseline health status. Baseline health status was defined as which of the three categories the patient was in at enrollment (i.e., ICU; hospitalized, not in ICU, on oxygen; and hospitalized, not in ICU, not on oxygen). For the win ratio, we stratified comparisons between randomized groups according to baseline health status.

**Table 1:** Models fitted to endpoints for the simulated data.

Model	Endpoint	Endpoint Variable Type	Coefficient Interpretation
Simple Logistic	E1: Proportion of patients hospitalized or dead on day 7	Binary	Odds ratio of discharged from the hospital versus not discharged on day 7
Sliding Dichotomy	E2: Proportion of patients moving to less severe categories from day 0 to day 7	Binary	Odds ratio of moving versus not moving to a less severe category from day 0 to day 7
Win Ratio	E3: Winners versus losers between IVIG and placebo on day 7	Binary	For all possible comparisons of patients in IVIG versus placebo, the number of IVIG winners divided by the number of IVIG losers
Proportional Odds^a^	E4: Day 7 ordinal endpoint	Ordinal	Average odds ratio of being in a less versus more severe category on day 7
Longitudinal Ordinal Outcome	E5: Distribution of the ordinal endpoint over the seven days of follow-up	Ordinal Longitudinal	Average multiplicative increase in the odds ratio of being in a less versus more severe category across the follow-up period
Cox Proportional Hazards	E6: Number of days to first hospital discharge	Time-to-Event	Hazard ratio of time-to-hospital discharge
Accelerated Failure Time (Exponential^a^ and Weibull distributions)	E6: Number of days to first hospital discharge	Time-to-Event	Reduction in quantiles of time-to-hospital discharge

a Model was used in the analytic setting, displayed in Fig. 1, Fig. 2.

To generate longitudinal data comparable to the data that was expected in the FLU-IVIG trial, we used data from a cohort study of patients hospitalized with influenza at many of the same sites participating in FLU-IVIG [17,18]. As of September 1st, 2016, the responses of the cohort study at randomization and across the follow-up period who met the trial eligibility criteria are given in Table 2. We used these longitudinal data to estimate category percentages for the placebo group of our study. Table 3 gives the category percentages of the ordinal endpoint at day 7 for the placebo and IVIG groups under proportional odds, as well as the five odds ratios for the five better versus worse divisions of the ordinal endpoint. A demonstration of how to derive the five odds ratios is included as a section at the end of this paper.

**Table 2:** FLU-IVIG placebo group distribution of the ordinal endpoint at randomization and across the 7 day follow-up period according to the cohort study [17,18].

Day of Evaluation	Death	ICU	Hospitalized, not in ICU, on oxygen	Hospitalized, not in ICU, not on oxygen	Discharged, not back to normal activities	Discharged, back to normal activities
Day 0 (%)^a^	0	7.6	46.1	46.3	0	0
Day 1 (%)	0.2	6.4	40.9	43.1	8.1	1.2
Day 2 (%)	0.2	6.7	33.7	37.9	18.5	3.0
Day 3 (%)	0.2	6.7	28.8	33.0	23.2	8.1
Day 4 (%)	0.5	6.4	21.2	26.8	31.8	13.3
Day 5 (%)	0.5	5.9	19.2	21.9	33.3	19.2
Day 6 (%)	1.0	4.9	17.7	18.5	34.7	23.2
Day 7 (%)	1.0	4.9	16.3	14.5	36.2	27.1

a (%) percentage of patients in the placebo group for the given ordinal endpoint category.

**Table 3:** Distributions of the FLU-IVIG placebo group and IVIG group on day 7 of follow-up. The FLU-IVIG placebo group distribution was estimated from the cohort study [17,18].

	Death	ICU	Hospitalized, not in ICU, on oxygen	Hospitalized, not in ICU, not on oxygen	Discharged, not back to normal activities	Discharged, back to normal activities
% Placebo^a^	1.0	4.9	16.3	14.5	36.2	27.1
% IVIG^b^	0.6	2.9	10.4	10.8	35.6	39.7
Odds ratio^c^		1.77	1.77	1.77	1.77	1.77

a % Placebo: percentage of patients in the placebo group for the given ordinal endpoint category.

b % IVIG: percentage of patients in the IVIG group for the given ordinal endpoint category.

c Odds ratio: The odds of having the given ordinal endpoint category or less severe versus more severe between the IVIG and placebo groups.

For the simulation, we first randomly sampled 320 patients from the day 0 distribution of the cohort study to create day 0 data for both groups (i.e., no treatment effect at randomization). We generated longitudinal data using a discrete-time Markov model whereby the day-to-day transition probabilities for the placebo group were estimated from the cohort study. For the treatment group, we varied on which days and for which groups of patients (e.g., hospitalized or dead versus discharged) the transition probabilities differed from the placebo group. Using a method from Peterson et al. [7], data were generated such that the average odds ratio on day 7 for the ordinal endpoint approximated 1.77, the pre-specified value of FLU-IVIG. Additionally, we varied the transition probabilities for the placebo group to simulate different target populations. Unlike the analytic setting, the proportional odds assumption may not hold on day 7 and time-to-first hospital discharge may not be exponentially distributed. The supplementary material explains the data generating process in greater detail.

We ran 10,000 simulations of the clinical trial for each treatment effect and placebo group combination. For each simulated trial, we fitted the seven models to the corresponding six endpoints and computed the corresponding Wald test statistics for the treatment effect. For each model, the empirical power is the proportion of the 10,000 simulations for which the Wald test statistic meets significance. Code to run our simulation in the R programming language can be downloaded from GitHub (https://github.com/RPeterson4/Comparative_FLU_IVIG_Code).

## 3 Results

The results section is divided into two parts. First, we analytically compare the ordinal endpoint evaluated at day 7 with the time-to-event endpoint evaluated at days 7–14. Second, we compare the ordinal endpoint by simulation to time-to-event, longitudinal, and binary endpoints.

### 3.1 Analytic comparison of ordinal endpoint to time-to-event endpoint

Fig. 1 compares the power of the ordinal endpoint to the time-to-event endpoint as a function of the granularity of the ordinal endpoint (i.e., $1-\overset{k}{\underset{i=1}{\sum }}{\bar{q}}_{i}^{3}$), the number of days of follow-up for the time-to-event endpoint, and the probability of discharge by day 7 in the placebo group ($p_{07}$). For fixed $p_{07}$, the time-to-event endpoint tends to perform better with additional days of follow-up. For $p_{07}$ values of 0.10 or lower and 0.63 or higher, ordinal endpoints of high granularity with values of 0.9 or higher for $1-\overset{k}{\underset{i=1}{\sum }}{\bar{q}}_{i}^{3}$ have an almost universal advantage over the time-to-event endpoint, including for the parameter values specified in the power calculations of the FLU-IVIG trial. For $p_{07}$ values ranging from 0.20 to 0.50, longer follow-up periods (i.e., from 8 to 14 days) grant the time-to-event endpoint a near universal advantage over the ordinal endpoint.

**Fig. 1:**
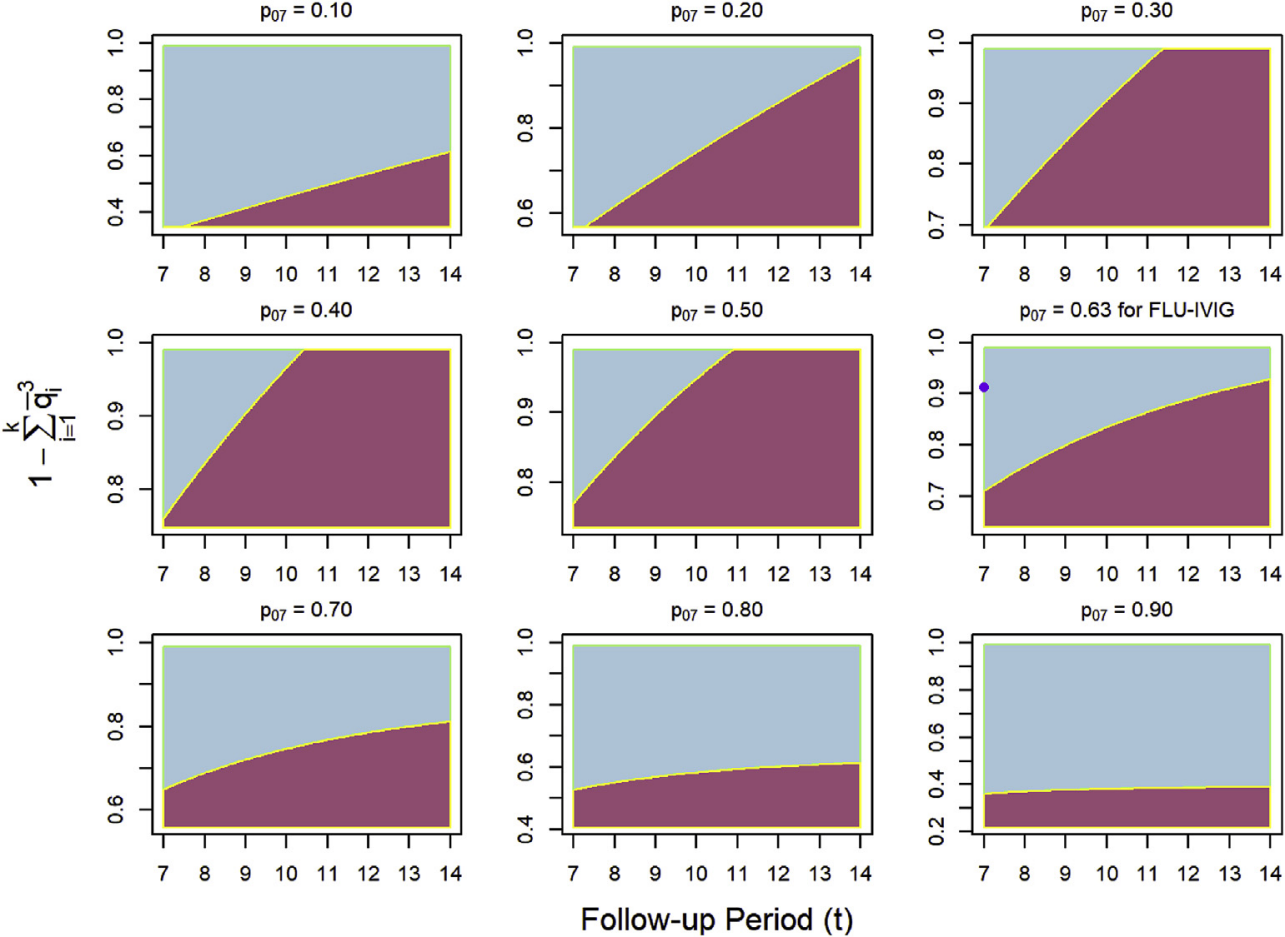
Comparison of the ordinal endpoint and time-to-event endpoint by follow-up period and $1-\overset{k}{\underset{i=1}{\sum }}{\bar{q}}_{i}^{3}$ according to their respective non-centrality parameters which uniquely determine power. Plots are reproduced across different probabilities of discharge by day 7 ($p_{07}$) for the placebo group. The ordinal endpoint is evaluated on day 7, while the time-to-event endpoint is evaluated across days 7–14. The blue region indicates that the ordinal endpoint yielded higher power, while the red region indicates that the time-to-event endpoint yielded higher power. The purple dot in the right-center plot marks the value for FLU-IVIG. As we assume that hospital discharge constitutes at least one category of the ordinal endpoint, the y-axes are bounded below by $1-\;{\left(\frac{p_{07}+p_{17}}{2}\right)}^{3}$. (For interpretation of the references to colour in this figure legend, the reader is referred to the Web version of this article.)

**Fig. 2:**
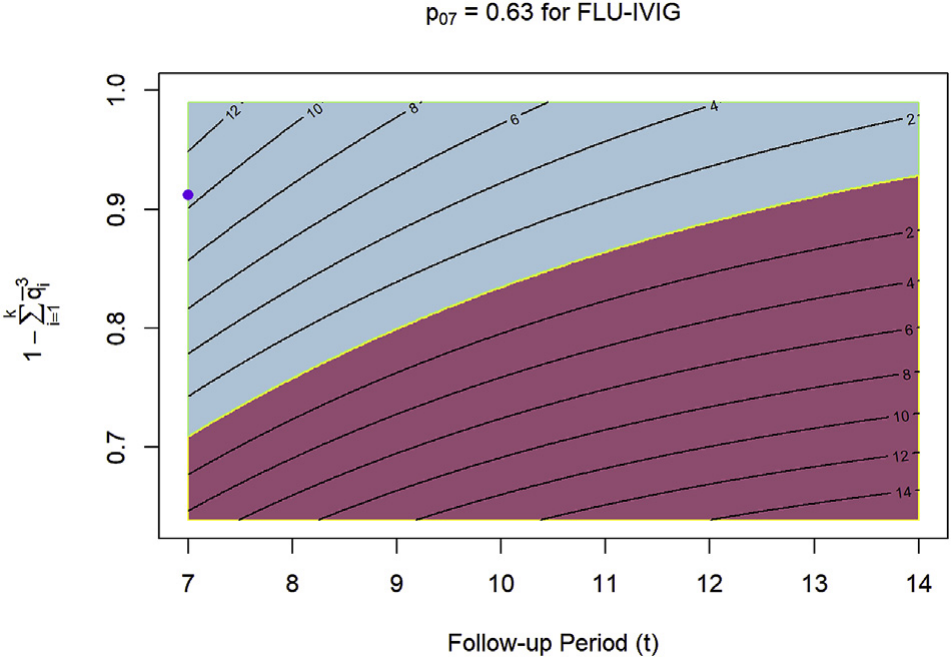
The right-center plot of Fig. 1 for FLU-IVIG with contours of power added to indicate by how much either the ordinal endpoint or the time-to-event endpoint surpassed the other in power. The blue region indicates that the ordinal endpoint yielded higher power, while the red region indicates that the time-to-event endpoint yielded higher power. The purple dot marks the value for FLU-IVIG. As we assume that hospital discharge constitutes at least one category of the ordinal endpoint, the y-axis is bounded below by $1-\;{\left(\frac{p_{07}+p_{17}}{2}\right)}^{3}$. (For interpretation of the references to colour in this figure legend, the reader is referred to the Web version of this article.)

Fig. 2 displays the right-center plot of Fig. 1 for FLU-IVIG but with contours of power added to further clarify the difference in performance between the time-to-event and ordinal endpoints. From day 7 to day 14 of follow-up, the time-to-event endpoint gains about 10% power over the ordinal endpoint. As the value of $1-\overset{k}{\underset{i=1}{\sum }}{\bar{q}}_{i}^{3}$ increases from the lower bound of $1-\;{\left(\frac{p_{07}+p_{17}}{2}\right)}^{3}$ to the upper bound of 1, the ordinal endpoint gains about 18% power over the time-to-event endpoint in that span.

### 3.2 Simulation comparison of ordinal endpoint to other endpoints

We investigated seven different treatment effects each with different transition probabilities across the seven days of follow-up. Each treatment effect is defined from days 1–7 of follow-up and arrives at an odds ratio of 1.77 on day 7:

• T1: The treatment effect remains constant for all categories across each day of follow-up.
• T2: The treatment effect only benefits patients for days 1–3 for all categories.
• T3: The treatment effect constantly decreases with each day with no additional benefit on day 7 for all categories.
• T4: The treatment effect remains constant across each day of follow-up but is 33.3% more effective for hospitalized patients on the log odds ratio scale.
• T5: The treatment effect only benefits patients in the “ICU” and “non-ICU, on oxygen categories” across each day of follow-up.
• T6: The treatment effect only benefits patients for days 5–7 for all categories.
• T7: The treatment effect constantly increases with each day, with benefit starting on day 2 for all categories.

We considered placebo group distributions corresponding to consistently more or less underlying risk throughout the follow-up period:

• The FLU-IVIG placebo group distribution based on the cohort study [17,18].
  o Category percentages on day 7 from death to hospital discharge with resumption of normal activities were: 1.0%, 4.9%, 16.3%, 14.5%, 36.2%, 27.1%.
• More severe placebo group (with more severe cases on each day of follow-up).
  o Category percentages on day 7 from death to hospital discharge with resumption of normal activities were: 2.0%, 9.3% 28.0%, 19.2%, 27.2%, 14.3%.
• Less severe group (with fewer severe cases on each day of follow-up).
  o Category percentages on day 7 from death to hospital discharge with resumption of normal activities were: 0.5%, 2.2%, 7.1%, 7.7%, 37.4%, 45.0%.

Supplementary Tables 1–3 display the respective placebo group distributions on day 7 and their corresponding IVIG group distributions for each treatment effect.

Table 4, Table 5, Table 6 give the estimated power for the six endpoints considered under different treatment effect scenarios and relative acuity in the patient population. Supplementary Tables 4–6 give the average coefficient values corresponding to each power estimate (for how to interpret each coefficient estimate, see Table 1). For the proportional odds model, adjusting for baseline health status (the bottom half of each table) universally raised power by about 4–9% points across all distributions of the placebo group and treatment effect scenarios considered (see Table 4, Table 5, Table 6). Across the three distributions of the placebo group, only the Cox and AFT models assuming a Weibull distribution yielded higher power for certain treatment effects (i.e., T2–4) relative to the proportional model, a finding that generally held with adjustment for baseline health status. Both time-to-event endpoints yielded low power under treatment effects T6 and T7 which assume delay of benefit. Note that the overall proportion of patients discharged by day 7 under each treatment effect scenario is relatively constant (see Supplementary Tables 1–3), but the discharge times in the treatment group differ significantly across scenarios. Overall, the Weibull AFT model performed better than the exponential AFT model.

**Table 4:** Simulated power (%)^a^ results for the FLU-IVIG placebo group on day 7 of follow-up ($p_{07}$^b^  = 0.63).^c^

Model	T1: Constant benefit^d^	T2: Benefit only for first three days	T3: Linear decrease in benefit	T4: Larger benefit for hospitalized patients	T5: Benefit only for ICU and non-ICU, on oxygen patients	T6: Benefit only for last three days	T7: Linear increase in benefit
No adjustment for baseline status							
Simple Logistic	71.65	75.07	75.29	76.70	83.20	60.79	67.87
Sliding Dichotomy	69.55	78.04	75.71	74.81	**93.04**	59.11	64.11
Win Ratio	**79.54**	79.58	79.38	79.61	79.66	**79.86**	**80.66**
Proportional Odds	**79.71**	79.69	79.45	79.68	79.86	**79.94**	**80.77**
Longitudinal Ordinal Outcome	**82.98**	71.52	77.96	**83.35**	67.21	**79.45**	**84.59**
Cox Proportional Hazards	76.73	**96.46**	**92.66**	**81.92**	87.47	36.39	53.81
Accelerated Failure Time (Exponential)	68.43	**94.24**	**89.17**	74.72	81.44	27.45	42.49
Accelerated Failure Time (Weibull)	78.83	**96.64**	**93.62**	**83.78**	**88.55**	37.09	55.83
Adjustment for baseline status							
Simple Logistic	78.70	81.24	82.28	83.68	86.08	68.84	75.55
Sliding Dichotomy	71.96	79.82	77.92	77.49	**93.29**	61.88	67.09
Win Ratio^e^	83.67	82.90	82.59	83.94	72.09	**86.26**	**86.14**
Proportional Odds	**86.25**	86.50	85.95	**86.50**	**87.26**	**86.93**	**86.70**
Longitudinal Ordinal Outcome	70.45	23.81	42.36	70.12	53.20	**84.90**	**85.63**
Cox Proportional Hazards	**84.93**	**98.92**	**96.89**	**89.27**	81.81	40.94	60.28
Accelerated Failure Time (Exponential)	74.41	**97.37**	**93.94**	80.50	75.69	26.67	43.51
Accelerated Failure Time (Weibull)	**86.23**	**98.98**	**97.21**	**90.48**	82.51	41.08	61.99

a Power (%): percentage of the 10,000 simulated datasets in which the Wald test statistic for the estimated treatment effect of the fitted model was significant at the two-sided 0.05 level. Power for the top three models for each treatment effect with or without adjustment for baseline health status is printed in bold.

b $p_{07}$ denotes the probability of discharge (i.e., categories 5 and 6 of the ordinal endpoint combined) by day 7 of follow-up for the placebo group.

c Supplementary Table 1 displays the category percentages for the FLU-IVIG placebo group on day 7 and each of the seven IVIG groups.

d Benefit refers to the differences in transition probabilities between randomized groups across treatment effects.

e Unlike all other models which include a covariate to adjust for baseline status, the win ratio stratifies by only comparing patients who started at the same baseline category between randomized groups.

**Table 5:** Simulated power (%)^a^ results for the more severe placebo group on day 7 of follow-up ($p_{07}$^b^  = 0.42).^c^

Model	T1: Constant benefit^d^	T2: Benefit only for first three days	T3: Linear decrease in benefit	T4: Larger benefit for hospitalized patients	T5: Benefit only for ICU and non-ICU, on oxygen patients	T6: Benefit only for last three days	T7: Linear increase in benefit
No adjustment for baseline status							
Simple Logistic	82.12	80.31	81.22	83.64	65.36	80.66	**82.11**
Sliding Dichotomy	**82.88**	86.93	85.24	**84.25**	**90.98**	79.40	80.78
Win Ratio	80.85	81.52	80.71	81.15	80.61	**81.28**	81.04
Proportional Odds	80.97	81.59	80.89	81.31	80.77	**81.43**	**81.21**
Longitudinal Ordinal Outcome	**91.73**	87.42	**90.20**	**92.16**	**87.42**	**85.37**	**90.88**
Cox Proportional Hazards	82.14	**92.36**	**89.57**	84.21	68.21	62.28	71.95
Accelerated Failure Time (Exponential)	78.48	**90.77**	87.13	80.57	63.60	56.69	67.01
Accelerated Failure Time (Weibull)	**82.70**	**92.53**	**90.08**	**84.90**	68.96	61.33	72.09
Adjustment for baseline status							
Simple Logistic	87.82	85.40	86.69	88.66	69.21	86.46	87.88
Sliding Dichotomy	85.13	88.85	87.48	86.52	**91.51**	82.00	83.25
Win Ratio^e^	**89.45**	88.78	88.41	88.64	**88.84**	**89.51**	**89.62**
Proportional Odds	**90.92**	91.00	90.35	**90.86**	**89.69**	**91.66**	**90.76**
Longitudinal Ordinal Outcome	84.67	49.41	65.97	84.07	82.32	**92.25**	**92.45**
Cox Proportional Hazards	88.46	**96.79**	**94.64**	**90.08**	64.76	66.60	77.72
Accelerated Failure Time (Exponential)	83.84	**95.45**	**92.43**	86.31	60.35	57.61	70.32
Accelerated Failure Time (Weibull)	**88.92**	**96.85**	**94.92**	**90.61**	65.07	65.65	77.73

a Power (%): percentage of the 10,000 simulated datasets in which the Wald test statistic for the estimated treatment effect of the fitted model was significant at the two-sided 0.05 level. Power for the top three models for each treatment effect with or without adjustment for baseline health status is printed in bold.

b $p_{07}$ denotes the probability of discharge (i.e., categories 5 and 6 of the ordinal endpoint combined) by day 7 of follow-up for the placebo group.

c Supplementary Table 2 displays the category percentages for the more severe placebo group on day 7 and each of the seven IVIG groups.

d Benefit refers to the differences in transition probabilities between randomized groups across treatment effects.

e Unlike all other models which include a covariate to adjust for baseline status, the win ratio stratifies by only comparing patients who started at the same baseline category between randomized groups.

**Table 6:** Simulated power (%)^a^ results for the less severe placebo group on day 7 of follow-up ($p_{07}$^b^  = 0.82).^c^

Model	T1: Constant benefit^d^	T2: Benefit only for first three days	T3: Linear decrease in benefit	T4: Larger benefit for hospitalized patients	T6: Benefit only for last three days	T7: Linear increase in benefit
No adjustment for baseline status						
Simple Logistic	54.07	63.20	63.42	62.44	36.23	45.35
Sliding Dichotomy	47.75	58.77	56.92	55.50	32.77	39.06
Win Ratio	**75.87**	76.15	76.16	76.08	**76.09**	**75.59**
Proportional Odds	**75.90**	76.20	76.16	**76.13**	**76.11**	**75.63**
Longitudinal Ordinal Outcome	64.44	44.81	52.52	62.78	**65.88**	**70.26**
Cox Proportional Hazards	70.74	**99.04**	**95.81**	**80.04**	15.94	31.88
Accelerated Failure Time (Exponential)	55.18	**97.11**	**90.71**	66.84	7.51	17.25
Accelerated Failure Time (Weibull)	**75.21**	**99.11**	**96.52**	**83.48**	18.67	37.27
Adjustment for baseline status						
Simple Logistic	59.39	68.44	69.06	68.29	41.08	50.68
Sliding Dichotomy	49.97	60.64	58.93	57.89	34.40	41.08
Win Ratio^e^	73.82	72.61	72.31	73.28	**77.76**	**75.46**
Proportional Odds	**80.52**	81.15	80.80	**80.68**	**80.12**	**79.94**
Longitudinal Ordinal Outcome	45.15	6.13	14.09	40.69	71.35	70.70
Cox Proportional Hazards	**79.02**	**99.79**	**98.50**	**87.80**	17.60	37.29
Accelerated Failure Time (Exponential)	58.68	**98.82**	**94.48**	70.81	5.83	16.23
Accelerated Failure Time (Weibull)	**82.50**	**99.79**	**98.80**	**90.09**	20.03	41.97

Due to the skewness of the less severe placebo group distribution, a treatment effect corresponding to T5 that approximated an odds ratio of 1.77 on day 7 could not be found.

a Power (%): percentage of the 10,000 simulated datasets in which the Wald test statistic for the estimated treatment effect of the fitted model was significant at the two-sided 0.05 level. Power for the top three models for each treatment effect with or without adjustment for baseline health status is printed in bold.

b $p_{07}$ denotes the probability of discharge (i.e., categories 5 and 6 of the ordinal endpoint combined) by day 7 of follow-up for the placebo group.

c Supplementary Table 3 displays the category percentages for the less severe placebo group on day 7 and each of the six IVIG groups.

d Benefit refers to the differences in transition probabilities between randomized groups across treatment effects.

e Unlike all other models which include a covariate to adjust for baseline status, the win ratio stratifies by only comparing patients who started at the same baseline category between randomized groups.

Excluding the Cox and AFT models, the proportional odds model generally yielded the highest power across all treatment effects and distributions of the placebo group considered, including after adjustment for baseline health status (see Table 4, Table 5, Table 6). Moreover, the proportional odds model with adjustment for baseline health status consistently returned power close the pre-specified level of 80% or higher, ranging from 79.94% to 91.66%. No other model was able to maintain power at the desired level of 80% across all treatment effects and placebo group distributions. However, for each treatment effect under the more severe placebo group distribution, the longitudinal ordinal outcome and sliding dichotomy models generally yielded greater power than the proportional odds model (see Table 5). These advantages mostly failed to hold after adjustment for baseline health status. Across all treatment effects and placebo group distributions, the simple logistic model and win ratio generally yielded lower power relative to the proportional odds model including after adjustment for baseline health status.

## 4 Discussion

To our knowledge, FLU-IVIG is the first influenza trial to use an ordinal scale of patient outcomes as the primary endpoint. A variation of the ordinal scale is now being used by other influenza trials as a primary or secondary endpoint [5,6]. Any novel endpoint should be rigorously evaluated to address clinical and statistical concerns, especially one that makes use of a relatively uncommon data type like an ordinal scale. In particular, a novel endpoint should be both interpretable and able to more consistently detect a treatment effect relative to other endpoints that may be derived from the same information. In that regard, we compared the power of the FLU-IVIG ordinal endpoint to a time-to-event endpoint analytically and by simulation and to five other endpoints by simulation. Selecting the most efficient endpoint for a trial includes weighing a number of clinical and statistical factors, including the nature of the treatment effect, length of the follow-up period, and the target population's event rate of outcomes and gradation and severity of patient illness. Our case study of the FLU-IVIG ordinal endpoint helps contextualize the relative importance of these factors.

Provided that the hazard ratio remains constant over the follow-up period, we demonstrated analytically that time-to-event endpoints assessed over a longer follow-up period yield greater power than ordinal endpoints when given moderate placebo group discharge probabilities at the time the ordinal endpoint was assessed on day 7. With a high number of placebo group patients on the cusp of hospital discharge on day 7, we would expect the time-to-event endpoint to substantially improve in power with follow-up periods of longer than 7 days which provide more time for events (i.e., hospital discharge) to occur. Conversely, with high discharge probabilities on day 7 and hence more patients who have already had the event in the placebo group, we would expect longer follow-up periods to only marginally benefit the time-to-event endpoint. Similarly, with very low discharge probabilities on day 7, very few patients would be close to leaving the hospital in the placebo group. Follow-up periods beyond 14 days would then be required to raise the power of the time-to-event endpoint above the ordinal endpoint. If trial designers expect a fairly ill target population and can assume that the treatment effect will be constant over a prolonged period, time-to-event endpoints may be preferable.

Of course, a constant treatment effect may not be reasonable to assume. Our simulation study gives insight into the types of endpoints that may provide the highestpower under plausible expressions of a non-constant treatment effect. Regardless of the underlying risk in the placebo group, for treatment effects T2–4, the Cox and Weibull AFT models generally returned larger power relative to the proportional odds model with or without adjustment for baseline health status. This is likely because treatment effects T2–4 assume disproportionate benefit soon after randomization or to hospitalized patients to reduce time-to-hospital discharge. Conversely, when the treatment effect was delayed as in T6 and T7, the time-to-event endpoints performed relatively poorly. This is likely because treatment effects T6 and T7 fail to discharge patients from the hospital early on in follow-up. In terms of consistency, the proportional odds model with adjustment for baseline health status was the only model to return power at the pre-specified level or higher across all treatment effect and placebo group combinations.

Alternatively, the ordinal endpoint evaluated on day 7 could be dichotomized into a binary endpoint but with the potential caveat of a loss of information. To that end, more complex binary endpoints have been proposed. For the purposes of our study, neither the win ratio, which compares ordinal endpoint category on day 7, nor the sliding dichotomy, which compares the change in patient health status between baseline and day 7, consistently improved power relative to the ordinal endpoint.

The win ratio without adjustment for baseline health status was always less than half a percentage point below the proportional odds model in power. With adjustment for baseline health status, the win ratio was often within a few percentage points of the proportional odds model. The gap may have widened because unlike all other models in this paper which include a covariate to adjust for baseline health status, the win ratio stratifies by only comparing patients who started at the same baseline category between randomized groups. This may have resulted in a number of ties in patient improvement to weaken increases in power. Overall, the win ratio closely tracked the proportional odds model in power and without adjustment for baseline health status may be preferable given its ease of interpretation relative to the odds ratio from fitting a proportional odds model.

In addition, longitudinal endpoints may perform better than endpoints assessed at a single time point. Yet, we found that the longitudinal ordinal outcome model was less powerful than evaluating the ordinal endpoint at a single time point, except when enrolling a target population with many hospitalized patients (i.e., the more severe placebo group). This may be because the longitudinal ordinal outcome model studied assumes a constant treatment effect over time, an erroneous assumption given our data generating mechanism in the simulation setting. Additionally, test statistics other than the Wald test statistic of the day by treatment group interaction may be more powerful.

In the simulation setting, our analysis found that adjusting for baseline health status universally increased power across all treatment effect and placebo group combinations for the proportional odds model, and for many of the other models. Analyses which include an adjustment for baseline health status should be considered even if the primary endpoint is not an ordinal endpoint.

Previous research has used Markov models to investigate infectious disease data in other areas including HIV [19,20]. In our case study of a severe influenza trial, we constructed transition matrices based off an ordinal endpoint to analytically derive placebo and IVIG groups with treatment effects that each approximated the pre-specified value of FLU-IVIG. Previous research has generally compared the proportional odds model to other models by fitting them to retrospective data [21]. Our simulation scheme provides a framework for how such data may be generated.

Our study is mainly limited by its specificity to the six-level ordinal endpoint and the type of target population considered in the FLU-IVIG trial. Of course, ordinal endpoints can be re-defined to better fit the target population. For example, if the FLU-IVIG ordinal endpoint were to be used for a more severely ill target population, the ICU category could be divided according to whether mechanical ventilation is required and the two hospital discharge categories could be combined. Additionally, in an unblinded study, the discharge categories as well as the oxygen categories could be combined due to their dependence on subjective clinician/patient assessments. Though different ordinal endpoints have been used in trials of vascular disease, *S. pneumoniae* infection, and traumatic brain injury [[21], [22], [23], [24]], our findings in the analytic setting and our general approach to evaluating the FLU-IVIG ordinal endpoint are applicable to those ordinal endpoints and others.

Overall, our findings suggest that ordinal endpoints with high granularity can reliably exceed time-to-event endpoints in power for hospitalized influenza populations in which most patients will naturally progress to hospital discharge by the time of endpoint assessment, similar to the FLU-IVIG trial. Furthermore, we find that the FLU-IVIG ordinal endpoint can perform even better after adjusting for baseline health status. However, if only 20–50% of patients will be discharged from the hospital by the end of the follow-up period, leaving many on the cusp of discharge, a time-to-event endpoint with a longer follow-up period may be more efficient. Additionally, a time-to-event endpoint may be able to more reliably detect a treatment effect that is strongest in the first few days following randomization.

More broadly, when deciding between an ordinal endpoint and a time-to-event endpoint, important factors to consider for each are the granularity of the ordinal endpoint and the follow-up period of the time-to-event endpoint. Categories which contain the largest proportions of patients should be divided as evenly as possible; conversely, categories which contain the fewest patients should be collapsed into categories with greater numbers. For the time-event endpoint, longer follow-up periods should be weighed to allow more time for events to occur. Moreover, longer follow-up periods and trials that cannot be blinded may not be ideal for ordinal endpoints that rely on patients to report information, like the two discharged categories in the FLU-IVIG ordinal endpoint. Concentrating on these two factors –granularity and follow-up period– in addition to the target population should help trial designers choose between ordinal endpoints and time-to-event endpoints for trials in severe influenza and other disease areas.

Although the choice of an ordinal outcome as the primary endpoint may be uncommon in clinical trials, our findings from this paper along with our previous paper and others provide strong support for the robustness of an ordinal endpoint to detect a treatment effect in trials of severe influenza [7,[21], [22], [23], [24]]. The data type of an ordinal scale with its multiple qualitative categories should not be discouraging to trial designers; rather, if an ordinal scale is to be considered, factors that may influence its clinical relevance and power should be thoroughly investigated.

## Derivation of the five odds ratios

Under the proportional odds assumption of the model, the odds ratio of having a given category or less severe versus more severe for any binary split of the ordinal endpoint is fixed at 1.77. For example, for the binary split of discharged, back to normal activities versus discharged, not back to normal activities or worse, the odds ratio can be derived as:

$$\frac{39.7/\left(1-39.7\right)}{27.1/\left(1-27.1\right)}=1.77$$

Similarly, for the binary split of discharged versus not discharged or dead, the odds ratio can be derived as:

$$\frac{\left(39.7+35.6/(1-39.7-35.6\right)}{\left(27.1+36.2)/(1-27.1-36.2\right)}=1.77$$

## Funding

Research reported in this publication was provided by subcontract 13XS134 under Leidos Biomed’s Prime Contract HHSN261200800001Eand HHSN2612015000031, NCI/NIAID/NIH and by NHLBI/NIH Award Number T32HL129956. Content is solely the responsibility of the authors and does not necessarily represent the views of the National Institutes of Health.

## Conflicts of interest

None declared.
