# Supplementary material for: Comparison of an ordinal endpoint to time-to-event, longitudinal, and binary endpoints for use in evaluating treatments for severe influenza requiring hospitalization
Source: Contemp Clin Trials Commun. 2019 Jun 21;15:100401. doi: 10.1016/j.conctc.2019.100401 (PMC6609815; doi:10.1016/j.conctc.2019.100401)
Supplement: Multimedia component 1 [file mmc1.docx]

**Supplementary Material**

**Derivation of the non-centrality parameter for the accelerated failure time model**

For the $i$th patient, assume that we have an endpoint based on the event of time to hospital discharge and define:

- $T_{i}$ as the time to hospital discharge with $T_{ip\delta}$ and $T_{it\delta}$ as the time to hospital discharge in the placebo and treatment groups, respectively.
- $A_{i}$ as randomized group status with $A_{i}=0$ indicating placebo group and $A_{i}=1$ indicating treatment group.
- $\delta_{p}$ and $\delta_{t}$ as the number of events (i.e., discharges) in the placebo and treatment groups, respectively.
- $c_{p}$ and $c_{t}$ as the number of censored observations in the placebo and treatment groups, respectively.

The parametric data distribution is then:

$$T_{i}|A_{i} \sim\mathrm{Exp}\left( e^{\gamma_{0}+\gamma_{1}}* e^{{-e}^{\gamma_{0}+ \gamma_{1} A_{i}}T_{i}} \right)^{\delta_{i}} \left( e^{{-e}^{\gamma_{0}+ \gamma_{1} A_{i}}T_{i}} \right)^{{1-\delta}_{i}}$$

Note that the time to censoring is fixed at 7. Then the log likelihood is:

$$\log L\left( \gamma_{0},\gamma_{1}|T_{1},A_{1},\ldots,T_{n},A_{n} \right)=\gamma_{0}\delta_{p}-e^{\gamma_{0}}\sum_{i=1}^{\delta_{p}} T_{ip\delta}+{(\gamma}_{0}+\gamma_{1})\delta_{t} {- e}^{\gamma_{0}+\gamma_{1}}\sum_{i=1}^{\delta_{t}} T_{it\delta}{- e}^{\gamma_{0}}7c_{p}{- e}^{\gamma_{0}+\gamma_{1}}7c_{t}$$

The Hessian matrix of the log likelihood with respect to $\gamma_{0}$ and $\gamma_{1}$ is derived as:

$\left( \begin{matrix} \frac{d^{2}}{d\gamma_{0}^{2}} & \frac{d^{2}}{d\gamma_{0}d\gamma_{1}} \\ \frac{d^{2}}{d\gamma_{0}d\gamma_{1}} & \frac{d^{2}}{d\gamma_{1}^{2}} \end{matrix} \right)= \left( \begin{matrix} -e^{\gamma_{0}}\sum_{i=1}^{\delta_{p}} T_{ip\delta}{- e}^{\gamma_{0}+\gamma_{1}}\sum_{i=1}^{\delta_{t}} T_{it\delta}{- e}^{\gamma_{0}}7c_{p}{- e}^{\gamma_{0}+\gamma_{1}}7c_{t} & {-e}^{\gamma_{0}+\gamma_{1}}\sum_{i=1}^{\delta_{t}} T_{it\delta}{- e}^{\gamma_{0}+\gamma_{1}}7c_{t} \\ {-e}^{\gamma_{0}+\gamma_{1}}\sum_{i=1}^{\delta_{t}} T_{it\delta}{- e}^{\gamma_{0}+\gamma_{1}}7c_{t} & {-e}^{\gamma_{0}+\gamma_{1}}\sum_{i=1}^{\delta_{t}} T_{it\delta}{- e}^{\gamma_{0}+\gamma_{1}}7c_{t} \end{matrix} \right)$

Focusing on the non-upper left terms of the Hessian, iterated expectation conditional on $\delta_{t}$ yields:

$${E(-e}^{\gamma_{0}+\gamma_{1}}\sum_{i=1}^{\delta_{t}} T_{it\delta}{- e}^{\gamma_{0}+\gamma_{1}}7c_{t})=E({E(-e}^{\gamma_{0}+\gamma_{1}}\sum_{i=1}^{\delta_{t}} T_{it\delta}{- e}^{\gamma_{0}+\gamma_{1}}7c_{t}|\delta_{t}))={-e}^{\gamma_{0}+\gamma_{1}}E\left( E\left( \sum_{i=1}^{\delta_{t}} T_{it\delta}|\delta_{t} \right) \right){- e}^{\gamma_{0}+\gamma_{1}}7E\left( c_{t} \right)={-e}^{\gamma_{0}+\gamma_{1}}E\left( \delta_{t}E\left( T_{it\delta} \right) \right){- e}^{\gamma_{0}+\gamma_{1}}7E(c_{t})=\frac{{-e}^{\gamma_{0}+\gamma_{1}}np_{1}E\left( T_{it\delta} \right)-e^{\gamma_{0}+\gamma_{1}}7n(1-p_{1})}{2}$$

where we have additionally made use of the fact that $\delta_{t}=n-c_{t}$, thus $E(\delta_{t})=n-{E(c}_{t})$. A similar argument can be used to simplify the upper left term of the Hessian.

The negative expected value of the Hessian is then:

$-E\left( \begin{matrix} \frac{d^{2}}{d\gamma_{0}^{2}} & \frac{d^{2}}{d\gamma_{0}d\gamma_{1}} \\ \frac{d^{2}}{d\gamma_{0}d\gamma_{1}} & \frac{d^{2}}{d\gamma_{1}^{2}} \end{matrix} \right)= \left( \begin{matrix} \frac{{e^{\gamma_{0}}np_{0}E\left( T_{ip\delta} \right) + e}^{\gamma_{0}+\gamma_{1}}np_{1}E\left( T_{it\delta} \right) +e^{\gamma_{0}}7n\left( 1-p_{0} \right)+e^{\gamma_{0}+\gamma_{1}}7n\left( 1-p_{1} \right)}{2} & \frac{e^{\gamma_{0}+\gamma_{1}}np_{1}E\left( T_{it\delta} \right) +e^{\gamma_{0}+\gamma_{1}}7n\left( 1-p_{1} \right)}{2} \\ \frac{e^{\gamma_{0}+\gamma_{1}}np_{1}E\left( T_{it\delta} \right) +e^{\gamma_{0}+\gamma_{1}}7n\left( 1-p_{1} \right)}{2} & \frac{e^{\gamma_{0}+\gamma_{1}}np_{1}E\left( T_{it\delta} \right) +e^{\gamma_{0}+\gamma_{1}}7n\left( 1-p_{1} \right)}{2} \end{matrix} \right)=\frac{{ne}^{\gamma_{0}}}{2}\left( \begin{matrix} {p_{0}E\left( T_{ip\delta} \right) + e}^{\gamma_{1}}p_{1}E\left( T_{it\delta} \right) +7\left( 1-p_{0} \right)+e^{\gamma_{1}}7\left( 1-p_{1} \right) & e^{\gamma_{1}}p_{1}E\left( T_{it\delta} \right) +e^{\gamma_{1}}7\left( 1-p_{1} \right) \\ e^{\gamma_{1}}p_{1}E\left( T_{it\delta} \right) +e^{\gamma_{1}}7\left( 1-p_{1} \right) & e^{\gamma_{1}}p_{1}E\left( T_{it\delta} \right) +e^{\gamma_{1}}7\left( 1-p_{1} \right) \end{matrix} \right)$

For the analytic calculation of power, we are only interested in the lower right element of the inverse of the Fisher’s information, derived as:

$$\frac{2}{{ne}^{\gamma_{0}}}\frac{{p_{0}E\left( T_{ip\delta} \right) + e}^{\gamma_{1}}p_{1}E\left( T_{it\delta} \right) +7\left( 1-p_{0} \right)+e^{\gamma_{1}}7\left( 1-p_{1} \right)}{e^{\gamma_{1}}(E\left( T_{ip\delta} \right)p_{0}-7p_{0}+7)(E\left( T_{it\delta} \right)p_{1}-7p_{1}+7)}= \frac{2}{{ne}^{\gamma_{0}}}\left( \frac{1}{E\left( T_{ip\delta} \right)p_{0}-7p_{0}+7}+\frac{1}{e^{\gamma_{1}}(E\left( T_{it\delta} \right)p_{1}-7p_{1}+7)} \right)$$

Note that:

$$E\left( T_{ip\delta} \right)=E\left( T_{i} | T_{i}<7,A_{i}=0 \right)= \frac{E\left( T_{i} | A_{i}=0 \right)-E\left( T_{i} \right|T_{I}>7,A_{i}=0)P(T_{i}>7 |A_{i}=0)}{P(T_{i}<7|A_{i}=0)}=\frac{e^{-\gamma_{0}}-\left( e^{-\gamma_{0}}+7 \right)(1-p_{0})}{p_{0}}$$

Similarly:

$$E\left( T_{it\delta} \right)=\frac{e^{-{(\gamma}_{0}+\gamma_{1})}-\left( e^{-{(\gamma}_{0}+\gamma_{1})}+7 \right)(1-p_{1})}{p_{1}}$$

Then:

$$\frac{2}{{ne}^{\gamma_{0}}}\left( \frac{1}{E\left( T_{ip\delta} \right)p_{0}-7p_{0}+7}+\frac{1}{e^{\gamma_{1}}(E\left( T_{it\delta} \right)p_{1}-7p_{1}+7)} \right)=\frac{2}{{ne}^{\gamma_{0}}}\left( \frac{e^{\gamma_{0}}}{p_{0}}+\frac{e^{\gamma_{0}}}{p_{1}} \right)=\frac{2}{n}\left( \frac{1}{p_{0}}+\frac{1}{p_{1}} \right)$$

Yielding the non-centrality parameter of:

$$c_{2}=log \left[ \frac{log(1-p_{1})}{log(1-p_{0})} \right]* \left[ \frac{2}{n}\left( \frac{1}{p_{0}}+\frac{1}{p_{1}} \right) \right]^{-1/2}$$

**In-depth explanation of longitudinal data generating process**

We used data from the cohort study to estimate the distribution of the placebo group. Data has been updated since the start of the FLU-IVIG trial. Supplementary Tables 1–3 display the distributions of the placebo and IVIG groups at day 7 across the different treatment effects and populations for the simulation setting. We used the placebo group cohort data to construct corresponding transition matrices between categories across days (see Supplementary Tables 7–13). Our choice of transition matrices makes use of the Markov property, in which each transition probability only depends on the category of the given day.

We used these transition matrices to generate IVIG longitudinal data. Each row of transition probabilities can be uniquely determined by its cumulative log odds of being in a more versus less severe category. Adding (subtracting) a constant to the cumulative log odds shifts the transition probabilities to favor more (less) severe categories. Thus, we subtracted constants from the cumulative log odds of each row of each transition matrix from the cohort study to construct potential transition matrices for the IVIG group. We used these transition matrices to analytically derive corresponding IVIG group distributions on day 7. A method devised by Peterson et al.^6^ can determine the corresponding odds ratio for the treatment effect.

Given that the FLU-IVIG trial pre-specified an odds ratio of 1.77, we found subtractive constants that yielded IVIG group distributions on day 7 with odds ratios within one one thousandth of 1.77. These subtractive constants made for the treatment effects examined in our paper. Details about the subtractive constants are provided in Supplementary Table 14.

To vary the placebo group distribution across days, we added or subtracted a constant to the cumulative log odds of the day 0 placebo group distribution as well as the rows of each transition matrix. Adding (subtracting) a constant yields analytic placebo group distributions on day 7 with more (fewer) severe cases. See Supplementary Tables 15 and 16 for respective examples of how to derive the cumulative log odds and the analytic placebo group distributions.

To create corresponding treatment effects for the more and less severe placebo groups, we subtracted constants from their respective transition matrices on top of what had been added or subtracted already. However, due to the skewness of the transition probabilities under P3, we were unable to find a subtractive constant for T5 that returned an odds ratio of approximately 1.77.

Note that for the FLU-IVIG placebo group, we were unable to find a subtractive constant that yielded an IVIG group distribution on day 7 that approximated proportional odds, a pre-specification of FLU-IVIG. However, our treatment effects for each placebo group only mildly deviate from proportional odds. For the FLU-IVIG placebo group, Table 3 displays the IVIG group distribution assuming that proportional odds holds.

**Supplementary Table 1.** Day 7 distributions of the ordinal endpoint for the FLU-IVIG placebo group (P1) and its corresponding treatment effects.

| Scenario | Death | ICU | Hospitalized, not in ICU, on oxygen | Hospitalized, not in ICU, not on oxygen | Discharged, not back to normal activities | Discharged, back to normal activities | Sum of discharged categories | Analytic odds ratio |
| --- | --- | --- | --- | --- | --- | --- | --- | --- |
| P1: FLU-IVIG Placebo Group (%)^a^ | 1.0 | 4.9 | 16.3 | 14.5 | 36.2 | 27.1 | 63.3 |  |
| T1: Constant benefit (%) | 0.6 | 3.6 | 10.3 | 9.2 | 37.0 | 39.3 | 76.3 | 1.770 |
| T2: Benefit only for first three days (%) | 0.7 | 3.1 | 9.3 | 10.0 | 38.8 | 38.1 | 76.9 | 1.771 |
| T3: Linear decrease in benefit (%) | 0.6 | 3.3 | 9.5 | 9.6 | 38.7 | 38.2 | 76.9 | 1.770 |
| T4: Larger benefit for hospitalized patients (%) | 0.6 | 3.5 | 9.9 | 9.0 | 38.4 | 38.6 | 77.0 | 1.770 |
| T5: Benefit only for In ICU and non-ICU, on oxygen patients (%) | 0.7 | 1.9 | 4.0 | 15.4 | 44.7 | 33.3 | 78.0 | 1.769 |
| T6: Benefit only for last three days (%) | 0.7 | 3.9 | 11.6 | 8.8 | 33.3 | 41.6 | 74.9 | 1.770 |
| T7: Linear increase in benefit (%) | 0.7 | 3.8 | 10.9 | 8.9 | 35.2 | 40.5 | 75.7 | 1.771 |

^a^(%) percentage of patients in the placebo group for the given ordinal endpoint category.

The placebo group distribution in our study has been updated from the FLU-IVIG protocol using data from the cohort study. For reference, the category percentages in the FLU-IVIG protocol are 1.8, 3.6, 15.6, 14.1, 39.0, and 25.8% for death through discharged, back to normal activities, respectively.

**Supplementary Table 2.** Day 7 distributions of the ordinal endpoint for the more severe placebo group (P2) and its corresponding treatment effects.

| Scenario | Death | ICU | Hospitalized, not in ICU, on oxygen | Hospitalized, not in ICU, not on oxygen | Discharged, not back to normal activities | Discharged, back to normal activities | Sum of discharged categories | Analytic odds ratio |
| --- | --- | --- | --- | --- | --- | --- | --- | --- |
| P2: More severe placebo group (%)^a^ | 2.0 | 9.3 | 28.0 | 19.2 | 27.2 | 14.3 | 41.5 |  |
| T1: Constant benefit (%) | 1.2 | 7.3 | 19.6 | 14.4 | 33.6 | 23.9 | 57.5 | 1.769 |
| T2: Benefit only for first three days (%) | 1.5 | 6.5 | 18.8 | 16.2 | 34.7 | 22.4 | 57.1 | 1.770 |
| T3: Linear decrease in benefit (%) | 1.3 | 6.9 | 18.9 | 15.5 | 34.8 | 22.6 | 57.4 | 1.770 |
| T4: Larger benefit for hospitalized patients (%) | 1.2 | 7.2 | 19.4 | 14.4 | 34.7 | 23.2 | 57.9 | 1.770 |
| T5: Benefit only for In ICU and non-ICU, on oxygen patients (%) | 1.3 | 6.1 | 14.1 | 23.7 | 36.2 | 18.5 | 54.7 | 1.770 |
| T6: Benefit only for last three days (%) | 1.3 | 7.7 | 20.9 | 12.7 | 30.4 | 26.9 | 57.3 | 1.770 |
| T7: Linear increase in benefit (%) | 1.3 | 7.6 | 20.1 | 13.4 | 32.2 | 25.4 | 57.6 | 1.770 |

^a^(%) percentage of patients in the placebo group for the given ordinal endpoint category.

**Supplementary Table 3.** Day 7 distributions of the ordinal endpoint for the less severe placebo group (P3) and its corresponding treatment effects.

| Scenario | Death | ICU | Hospitalized, not in ICU, on oxygen | Hospitalized, not in ICU, not on oxygen | Discharged, not back to normal activities | Discharged, back to normal activities | Sum of discharged categories | Analytic odds ratio |
| --- | --- | --- | --- | --- | --- | --- | --- | --- |
| P3: Less severe placebo group (%)^a^ | 0.5 | 2.2 | 7.1 | 7.7 | 37.4 | 45.0 | 82.4 |  |
| T1: Constant benefit (%) | 0.3 | 1.5 | 3.9 | 4.0 | 31.7 | 58.6 | 90.3 | 1.770 |
| T2: Benefit only for first three days (%) | 0.3 | 1.2 | 3.3 | 4.1 | 33.1 | 58.0 | 91.3 | 1.770 |
| T3: Linear decrease in benefit (%) | 0.3 | 1.3 | 3.4 | 4.0 | 33.1 | 58.0 | 91.1 | 1.769 |
| T4: Larger benefit for hospitalized patients (%) | 0.3 | 1.4 | 3.6 | 3.7 | 32.9 | 58.1 | 91.0 | 1.770 |
| T6: Benefit only for last three days (%) | 0.3 | 1.7 | 4.8 | 4.4 | 29.2 | 59.6 | 88.8 | 1.770 |
| T7: Linear increase in benefit (%) | 0.3 | 1.6 | 4.4 | 4.2 | 30.4 | 59.1 | 89.5 | 1.770 |

^a^(%) percentage of patients in the placebo group for the given ordinal endpoint category.

Due to the skewness of the P3 placebo group distribution, a treatment effect corresponding to T5 that approximated an odds ratio of 1.77 on day 7 could not be found.

**Supplementary Table 4.** Average coefficient values across the 10,000 trials for the FLU-IVIG placebo group. See Table 1 for how to interpret each coefficient.

| Model | T1: Constant benefit | T2: Benefit only for first three days | T3: Linear decrease in benefit | T4: Larger benefit for hospitalized patients | T5: Benefit only for ICU and non-ICU, on oxygen patients | T6: Benefit only for last three days | T7: Linear increase in benefit |
| --- | --- | --- | --- | --- | --- | --- | --- |
| No adjustment for baseline status | | | | | | | |
| Simple Logistic | 1.88 | 1.93 | 1.94 | 1.96 | 2.08 | 1.74 | 1.82 |
| Sliding Dichotomy | 1.98 | 2.14 | 2.09 | 2.07 | 2.68 | 1.81 | 1.88 |
| Win Ratio | 1.64 | 1.64 | 1.64 | 1.65 | 1.65 | 1.64 | 1.65 |
| Proportional Odds | 1.77 | 1.77 | 1.77 | 1.78 | 1.78 | 1.78 | 1.78 |
| Longitudinal Ordinal Outcome | 1.10 | 1.08 | 1.09 | 1.10 | 1.09 | 1.09 | 1.10 |
| Cox Proportional Hazards | 1.43 | 1.65 | 1.57 | 1.47 | 1.51 | 1.24 | 1.32 |
| Accelerated Failure Time (Exponential) | 0.32 | 0.45 | 0.41 | 0.34 | 0.36 | 0.19 | 0.24 |
| Accelerated Failure Time (Weibull) | 0.24 | 0.37 | 0.32 | 0.26 | 0.27 | 0.13 | 0.17 |
| Adjustment for baseline status | | | | | | | |
| Simple Logistic | 2.12 | 2.18 | 2.20 | 2.23 | 2.24 | 1.95 | 2.05 |
| Sliding Dichotomy | 2.08 | 2.24 | 2.20 | 2.18 | 2.74 | 1.90 | 1.98 |
| Win Ratio | 1.78 | 1.76 | 1.76 | 1.78 | 1.63 | 1.81 | 1.80 |
| Proportional Odds | 1.91 | 1.91 | 1.91 | 1.91 | 1.93 | 1.92 | 1.91 |
| Longitudinal Ordinal Outcome | 1.11 | 1.05 | 1.07 | 1.11 | 1.09 | 1.13 | 1.13 |
| Cox Proportional Hazards | 1.49 | 1.77 | 1.67 | 1.53 | 1.47 | 1.27 | 1.35 |
| Accelerated Failure Time (Exponential) | 0.34 | 0.49 | 0.44 | 0.36 | 0.34 | 0.20 | 0.25 |
| Accelerated Failure Time (Weibull) | 0.25 | 0.39 | 0.34 | 0.27 | 0.24 | 0.13 | 0.17 |

**Supplementary Table 5.** Average coefficient values across the 10,000 trials for the more severe placebo group. See Table 1 for how to interpret each coefficient.

| Model | T1: Constant benefit | T2: Benefit only for first three days | T3: Linear decrease in benefit | T4: Larger benefit for hospitalized patients | T5: Benefit only for ICU and non-ICU, on oxygen patients | T6: Benefit only for last three days | T7: Linear increase in benefit |
| --- | --- | --- | --- | --- | --- | --- | --- |
| No adjustment for baseline status | | | | | | | |
| Simple Logistic | 1.91 | 1.88 | 1.90 | 1.94 | 1.70 | 1.89 | 1.92 |
| Sliding Dichotomy | 1.96 | 2.05 | 2.01 | 2.00 | 2.16 | 1.91 | 1.92 |
| Win Ratio | 1.61 | 1.61 | 1.61 | 1.61 | 1.61 | 1.61 | 1.61 |
| Proportional Odds | 1.77 | 1.78 | 1.77 | 1.78 | 1.77 | 1.78 | 1.78 |
| Longitudinal Ordinal Outcome | 1.11 | 1.10 | 1.10 | 1.11 | 1.10 | 1.10 | 1.11 |
| Cox Proportional Hazards | 1.58 | 1.71 | 1.66 | 1.60 | 1.48 | 1.44 | 1.50 |
| Accelerated Failure Time (Exponential) | 0.43 | 0.51 | 0.48 | 0.44 | 0.37 | 0.34 | 0.38 |
| Accelerated Failure Time (Weibull) | 0.30 | 0.41 | 0.37 | 0.31 | 0.26 | 0.21 | 0.24 |
| Adjustment for baseline status | | | | | | | |
| Simple Logistic | 2.16 | 2.11 | 2.14 | 2.20 | 1.81 | 2.15 | 2.18 |
| Sliding Dichotomy | 2.05 | 2.14 | 2.10 | 2.09 | 2.22 | 2.00 | 2.02 |
| Win Ratio | 1.85 | 1.84 | 1.84 | 1.85 | 1.82 | 1.86 | 1.86 |
| Proportional Odds | 1.96 | 1.97 | 1.96 | 1.97 | 1.95 | 1.99 | 1.97 |
| Longitudinal Ordinal Outcome | 1.12 | 1.07 | 1.10 | 1.12 | 1.12 | 1.14 | 1.14 |
| Cox Proportional Hazards | 1.65 | 1.83 | 1.76 | 1.68 | 1.46 | 1.48 | 1.55 |
| Accelerated Failure Time (Exponential) | 0.45 | 0.56 | 0.52 | 0.47 | 0.36 | 0.34 | 0.39 |
| Accelerated Failure Time (Weibull) | 0.31 | 0.43 | 0.39 | 0.32 | 0.24 | 0.21 | 0.24 |

**Supplementary Table 6.** Average coefficient values across the 10,000 trials for the less severe placebo group. See Table 1 for how to interpret each coefficient.

| Model | T1: Constant benefit | T2: Benefit only for first three days | T3: Linear decrease in benefit | T4: Larger benefit for hospitalized patients | T6: Benefit only for last three days | T7: Linear increase in benefit |
| --- | --- | --- | --- | --- | --- | --- |
| No adjustment for baseline status | | | | | | |
| Simple Logistic | 2.02 | 2.22 | 2.23 | 2.20 | 1.71 | 1.85 |
| Sliding Dichotomy | 2.20 | 2.52 | 2.47 | 2.41 | 1.83 | 1.98 |
| Win Ratio | 1.71 | 1.72 | 1.72 | 1.72 | 1.71 | 1.71 |
| Proportional Odds | 1.78 | 1.78 | 1.78 | 1.78 | 1.78 | 1.78 |
| Longitudinal Ordinal Outcome | 1.08 | 1.07 | 1.07 | 1.08 | 1.08 | 1.09 |
| Cox Proportional Hazards | 1.35 | 1.67 | 1.56 | 1.40 | 1.12 | 1.20 |
| Accelerated Failure Time (Exponential) | 0.25 | 0.43 | 0.37 | 0.28 | 0.10 | 0.15 |
| Accelerated Failure Time (Weibull) | 0.21 | 0.36 | 0.31 | 0.23 | 0.07 | 0.12 |
| Adjustment for baseline status | | | | | | |
| Simple Logistic | 2.29 | 2.52 | 2.55 | 2.52 | 1.88 | 2.07 |
| Sliding Dichotomy | 2.32 | 2.67 | 2.62 | 2.55 | 1.91 | 2.08 |
| Win Ratio | 1.79 | 1.77 | 1.77 | 1.77 | 1.83 | 1.81 |
| Proportional Odds | 1.87 | 1.89 | 1.88 | 1.87 | 1.88 | 1.87 |
| Longitudinal Ordinal Outcome | 1.09 | 1.01 | 1.04 | 1.08 | 1.12 | 1.12 |
| Cox Proportional Hazards | 1.40 | 1.79 | 1.65 | 1.46 | 1.13 | 1.22 |
| Accelerated Failure Time (Exponential) | 0.26 | 0.45 | 0.39 | 0.29 | 0.10 | 0.15 |
| Accelerated Failure Time (Weibull) | 0.21 | 0.38 | 0.33 | 0.24 | 0.07 | 0.12 |

Due to the skewness of the P3 placebo group distribution, a treatment effect corresponding to T5 that approximated an odds ratio of 1.77 on day 7 could not be found.

**Supplementary Table 7.** FLU-IVIG placebo group transition matrix from day 0 to day 1.

| Category on day 1 | | | | | |  |
| --- | --- | --- | --- | --- | --- | --- |
| Category on day 0 | Death | ICU | Hospitalized, not in ICU, on oxygen | Hospitalized, not in ICU, not on oxygen | Discharged, not back to normal activities | Discharged, back to normal activities |
| ICU | 0.032 | 0.839 | 0.065 | 0.065 | 0 | 0 |
| Hospitalized, not in ICU, on oxygen | 0 | 0 | 0.872 | 0.075 | 0.053 | 0 |
| Hospitalized, not in ICU, not on oxygen | 0 | 0 | 0.005 | 0.846 | 0.122 | 0.027 |

Each cell presents the probability of transitioning from the given row category on day 0 to the column category on day 1.

**Supplementary Table 8.** FLU-IVIG placebo group transition matrix from day 1 to day 2.

| Category on day 2 | | | | | |  |
| --- | --- | --- | --- | --- | --- | --- |
| Category on day 1 | Death | ICU | Hospitalized, not in ICU, on oxygen | Hospitalized, not in ICU, not on oxygen | Discharged, not back to normal activities | Discharged, back to normal activities |
| Death | 1 | 0 | 0 | 0 | 0 | 0 |
| ICU | 0 | 0.962 | 0.038 | 0 | 0 | 0 |
| Hospitalized, not in ICU, on oxygen | 0 | 0.006 | 0.807 | 0.096 | 0.084 | 0.006 |
| Hospitalized, not in ICU, not on oxygen | 0 | 0.006 | 0.011 | 0.783 | 0.183 | 0.017 |
| Discharged, not back to normal activities | 0 | 0 | 0 | 0.030 | 0.879 | 0.091 |
| Discharged, back to normal activities | 0 | 0 | 0 | 0 | 0 | 1 |

Each cell presents the probability of transitioning from the given row category on day 1 to the column category on day 2.

**Supplementary Table 9.** FLU-IVIG placebo group transition matrix from day 2 to day 3.

| Category on day 3 | | | | | |  |
| --- | --- | --- | --- | --- | --- | --- |
| Category on day 2 | Death | ICU | Hospitalized, not in ICU, on oxygen | Hospitalized, not in ICU, not on oxygen | Discharged, not back to normal activities | Discharged, back to normal activities |
| Death | 1 | 0 | 0 | 0 | 0 | 0 |
| ICU | 0 | 0.889 | 0.074 | 0.037 | 0 | 0 |
| Hospitalized, not in ICU, on oxygen | 0 | 0.007 | 0.839 | 0.117 | 0.29 | 0.007 |
| Hospitalized, not in ICU, not on oxygen | 0 | 0.013 | 0 | 0.760 | 0.130 | 0.097 |
| Discharged, not back to normal activities | 0 | 0 | 0 | 0 | 0.933 | 0.067 |
| Discharged, back to normal activities | 0 | 0 | 0 | 0 | 0 | 1 |

Each cell presents the probability of transitioning from the given row category on day 2 to the column category on day 3.

**Supplementary Table 10.** FLU-IVIG placebo group transition matrix from day 3 to day 4.

| Category on day 4 | | | | | |  |
| --- | --- | --- | --- | --- | --- | --- |
| Category on day 3 | Death | ICU | Hospitalized, not in ICU, on oxygen | Hospitalized, not in ICU, not on oxygen | Discharged, not back to normal activities | Discharged, back to normal activities |
| Death | 1 | 0 | 0 | 0 | 0 | 0 |
| ICU | 0 | 0.926 | 0 | 0.074 | 0 | 0 |
| Hospitalized, not in ICU, on oxygen | 0 | 0.009 | 0.726 | 0.111 | 0.137 | 0.017 |
| Hospitalized, not in ICU, not on oxygen | 0.007 | 0 | 0.007 | 0.694 | 0.187 | 0.104 |
| Discharged, not back to normal activities | 0 | 0 | 0 | 0.011 | 0.936 | 0.053 |
| Discharged, back to normal activities | 0 | 0 | 0 | 0 | 0 | 1 |

Each cell presents the probability of transitioning from the given row category on day 3 to the column category on day 4.

**Supplementary Table 11.** FLU-IVIG placebo group transition matrix from day 4 to day 5.

| Category on day 5 | | | | | |  |
| --- | --- | --- | --- | --- | --- | --- |
| Category on day 4 | Death | ICU | Hospitalized, not in ICU, on oxygen | Hospitalized, not in ICU, not on oxygen | Discharged, not back to normal activities | Discharged, back to normal activities |
| Death | 1 | 0 | 0 | 0 | 0 | 0 |
| ICU | 0 | 0.923 | 0.077 | 0 | 0 | 0 |
| Hospitalized, not in ICU, on oxygen | 0 | 0 | 0.884 | 0.035 | 0.070 | 0.012 |
| Hospitalized, not in ICU, not on oxygen | 0 | 0 | 0 | 0.761 | 0.119 | 0.119 |
| Discharged, not back to normal activities | 0 | 0 | 0 | 0.016 | 0.899 | 0.085 |
| Discharged, back to normal activities | 0 | 0 | 0 | 0.019 | 0 | 0.981 |

Each cell presents the probability of transitioning from the given row category on day 4 to the column category on day 5.

**Supplementary Table 12.** FLU-IVIG placebo group transition matrix from day 5 to day 6.

| Category on day 6 | | | | | |  |
| --- | --- | --- | --- | --- | --- | --- |
| Category on day 5 | Death | ICU | Hospitalized, not in ICU, on oxygen | Hospitalized, not in ICU, not on oxygen | Discharged, not back to normal activities | Discharged, back to normal activities |
| Death | 1 | 0 | 0 | 0 | 0 | 0 |
| ICU | 0.042 | 0.833 | 0.083 | 0.042 | 0 | 0 |
| Hospitalized, not in ICU, on oxygen | 0 | 0 | 0.885 | 0.090 | 0.013 | 0.013 |
| Hospitalized, not in ICU, not on oxygen | 0 | 0 | 0.011 | 0.753 | 0.191 | 0.045 |
| Discharged, not back to normal activities | 0.007 | 0 | 0 | 0 | 0.911 | 0.081 |
| Discharged, back to normal activities | 0 | 0 | 0 | 0 | 0 | 1 |

Each cell presents the probability of transitioning from the given row category on day 5 to the column category on day 6.

**Supplementary Table 13.** FLU-IVIG placebo group transition matrix from day 6 to day 7.

| Category on day 7 | | | | | |  |
| --- | --- | --- | --- | --- | --- | --- |
| Category on day 6 | Death | ICU | Hospitalized, not in ICU, on oxygen | Hospitalized, not in ICU, not on oxygen | Discharged, not back to normal activities | Discharged, back to normal activities |
| Death | 1 | 0 | 0 | 0 | 0 | 0 |
| ICU | 0 | 1 | 0 | 0 | 0 | 0 |
| Hospitalized, not in ICU, on oxygen | 0 | 0 | 0.917 | 0.056 | 0.014 | 0.014 |
| Hospitalized, not in ICU, not on oxygen | 0 | 0 | 0 | 0.733 | 0.213 | 0.053 |
| Discharged, not back to normal activities | 0 | 0 | 0 | 0 | 0.922 | 0.078 |
| Discharged, back to normal activities | 0 | 0 | 0 | 0 | 0 | 1 |

Each cell presents the probability of transitioning from the given row category on day 6 to the column category on day 7.

**Supplementary Table 14.** Mathematical descriptions of treatment effects designed to simulate potential IVIG data.

| Treatment effect | Descriptions of constants subtracted from the cumulative log odds of the rows of given placebo group transition matrices | Values of constants subtracted from the cumulative log odds | | |
| --- | --- | --- | --- | --- |
|  |  | P1: FLU-IVIG | P2: More Severe | P3: Less Severe |
| T1: The treatment effect remains constant for all categories across each day of follow-up. | Subtract the same constant from each row of all transition matrices | 0.411 | 0.475 | 0.377 |
| T2: The treatment effect only benefits patients for the first three days following randomization for all categories. | Subtract the same constant from each row of the first three transition matrices | 0.910 | 0.979 | 0.891 |
| T3: The treatment effect constantly decreases with each day with no additional benefit on day 7 for all categories. | Linear decrease in subtractive constant to all rows of each transition matrix. First day subtracts a constant, C, second days subtracts 5/6 C, third day subtracts 4/6 C, etc. No constant subtracted from the last transition matrix. | 0.808 | 0.882 | 0.787 |
| T4: The treatment effect remains constant across each day of follow-up but is 33.3% more effective for hospitalized patients on the log odds ratio scale. | For all transition matrices, subtract a constant, C, from each row corresponding to hospitalized patients. Subtract 3/4 C from row corresponding to discharged, not back to normal patients. | 0.437 | 0.489 | 0.424 |
| T5: The treatment effect only benefits patients in the “In ICU” and “non-ICU, on oxygen categories” across each day of follow-up. | Subtract the same constant from the two rows corresponding to In ICU and non-ICU, on oxygen patients for all transition matrices. | 1.100 | 0.834 | N/A |
| T6: The treatment effect only benefits patients for the last three days of follow-up for all categories. | Subtract the same constant from each row of the last three transition matrices. | 0.852 | 1.057 | 0.716 |
| T7: The treatment effect constantly increases with each day, with benefit starting on day 2 for all categories. | Linear increase in subtractive constant to all rows of each transition matrix. Second day subtracts 1/6 of a constant C, third day subtracts 2/6 C, fourth day subtracts 3/6 C, etc. No constant subtracted from the first transition matrix. | 0.764 | 0.922 | 0.666 |

All treatment effects return analytic IVIG distributions on day 7 that yield analytic odds ratios within one one thousandth of 1.77, the pre-specified value of FLU-IVIG.

Due to the skewness of the P3 placebo group distribution, a treatment effect corresponding to T5 that approximated an odds ratio of 1.77 on day 7 could not be found.

**Supplementary Table 15.** Altering the distribution of the day 0 placebo group to be more or less skewed by changing its cumulative log odds.

| Day 0 Placebo Group | | Death | ICU | Hospitalized, not in ICU, on oxygen | Hospitalized, not in ICU, not on oxygen | Discharged, not back to normal activities | Discharged, back to normal activities |
| --- | --- | --- | --- | --- | --- | --- | --- |
| P1: FLU-IVIG Placebo Group | % Placebo^a^  Cumulative log odds^b^ | 0 | 7.6  -2.49 | 46.1  0.15 | 46.3 | 0 | 0 |
| P2: More severe placebo group | % Placebo  Cumulative log odds | 0 | 12.0  -1.99 | 53.7  0.65 | 34.3 | 0 | 0 |
| P3: Less severe placebo group | % Placebo  Cumulative log odds | 0 | 4.8  -2.99 | 36.5  -0.35 | 58.7 | 0 | 0 |

^a^% Placebo: percentage of patients in the placebo group for the given ordinal endpoint category.

^b^Cumulative log odds: (natural) logarithm of the odds of the given category or more severe versus less severe.

**Derivation of the cumulative log odds for the distribution of the day 0 placebo group and the rows of each transition matrix**

For an ordinal endpoint $Y$, the cumulative log odds $C_{j}$ for level $j$ out of$J$ total levels in the distribution of the placebo group or a row of a transition matrix is:

$$\log\left( \frac{P(Y\leq j)}{P(Y>j)} \right)=C_{j} for j=1,2,\ldots J-1$$

To return to the cumulative probability, that is, $P(Y\leq j)$, use the expit function on $C_{j}$:

$$\frac{e^{C_{j}}}{1+ e^{C_{j}}}*100=P(Y\leq j)$$

The probability that $Y$ assumes level $j$, that is $P(Y=j)$, is then derived as:

$$P\left( Y=j \right)=P\left( Y\leq j \right)-P(Y\leq j-1)$$

A demonstration using patients in the ICU or Hospitalized, not in ICU, on oxygen for P1:

$$\log\left( \frac{7.6+46.1}{100-(7.6+46.1)} \right)=0.15$$

$$\frac{e^{0.15}}{1+ e^{0.15}}*100= 53.7=7.6+46.1$$

We also applied this method to the rows of the transition matrices of the FLU-IVIG placebo group to simulate the other placebo group transition matrices, as well as the IVIG transition matrices.

**Supplementary Table 16.** FLU-IVIG placebo group transition matrix from day 0 to day 1 additionally including distributions on both days.

| Category on day 1 | | | | | |  |  |
| --- | --- | --- | --- | --- | --- | --- | --- |
| Category on day 0 | Death | ICU | Hospitalized, not in ICU, on oxygen | Hospitalized, not in ICU, not on oxygen | Discharged, not back to normal activities | Discharged, back to normal activities | Day 0 (%)^a^ |
| ICU | 0.032 | 0.839 | 0.065 | 0.065 | 0 | 0 | 7.6 |
| Hospitalized, not in ICU, on oxygen | 0 | 0 | 0.872 | 0.075 | 0.053 | 0 | 46.1 |
| Hospitalized, not in ICU, not on oxygen | 0 | 0 | 0.005 | 0.846 | 0.122 | 0.027 | 46.3 |
| Day 1 (%) | 0.2 | 6.4 | 40.9 | 43.1 | 8.1 | 1.2 |  |

^a^(%) percentage of patients in the placebo group for the given ordinal endpoint category.

**Deriving analytic distributions of the ordinal endpoint for the placebo and IVIG groups**

Given the distribution of the ordinal endpoint on one day, we derived the analytic distribution of the ordinal endpoint on the next day by use of the corresponding transition matrix. Each next day category percentage can be calculated as the sum of the current day category percentages times the corresponding transition probabilities. That is:

$$P\left( next day category \right)=\sum_{j} P\left( current day category j \right)*P(move from current day category j to next day category)$$

For example, for the FLU-IVIG placebo group moving from day 0 to day 1:

Day 1 Death: 7.6 * 0.032 = 0.2

Day 1 ICU: 7.6 * 0.839 = 6.4

Day 1 Hospitalized, not in ICU, on oxygen: 7.6 * 0.065 + 46.1 * 0.872 + 46.3 * 0.005 = 40.9

Day 1 Hospitalized, not in ICU, not on oxygen: 7.6 * 0.065 + 46.1 * 0.075 + 0.463 * 0.846 = 43.1

Day 1 Discharged, not back to normal activities: 46.1 * 0.053 + 46.3 * 0.122 = 8.1

Day 1 Discharged, back to normal activities: 46.3 * 0.027 = 1.2

We cyclically applied this method to derive the analytic distribution of the ordinal endpoint on each following day. We also used this method to derive analytic distributions for the other placebo groups as well as the IVIG groups.
